# Supplementary material for: S100A9-CXCL12 activation in BRCA1-mutant breast cancer promotes an immunosuppressive microenvironment associated with resistance to immunotherapy
Source: Nat Commun. 2022 Mar 18;13:1481. doi: 10.1038/s41467-022-29151-5 (PMC8933470; doi:10.1038/s41467-022-29151-5)
Supplement: Supplementary file 1 — Supplementary Information [file 41467_2022_29151_MOESM1_ESM.pdf]

# Supplementary Information for

## S100A9-CXCL12 activation in BRCA1-Mutant Breast Cancer promotes an Immunosuppressive Microenvironment associated with Resistance to Immunotherapy

Jianjie Li [1,2], Xiaodong Shu[1,2], Jun Xu[1,2], Sek Man Su[1,2], Un In Chan[1,2], Lihua Mo[1,2], Jianlin Liu[1,2], Xin Zhang[1,2], Ragini Adhav[1,2], Qiang Chen[1,2], Yuqing Wang[1,2], Tingting An[1,2], Xu Zhang[1,2], Xueying Lyu [1,2], Xiaoling Li[1,2], Josh Haipeng Lei[1,2], Kai Miao[1,2,3] , Heng Sun[1,2,3], Fuqiang Xing[1,2], Aiping Zhang[1,2], Chuxia Deng[1,2,3] *, Xiaoling Xu[1,2,3] *

*Corresponding author: Chuxia Deng, Email: cxdeng@umac.mo, and Xiaoling Xu, Email: xiaolingx@umac.mo

**This PDF file includes:**

Supplementary Figures 1-9

Supplementary Tables 1-2

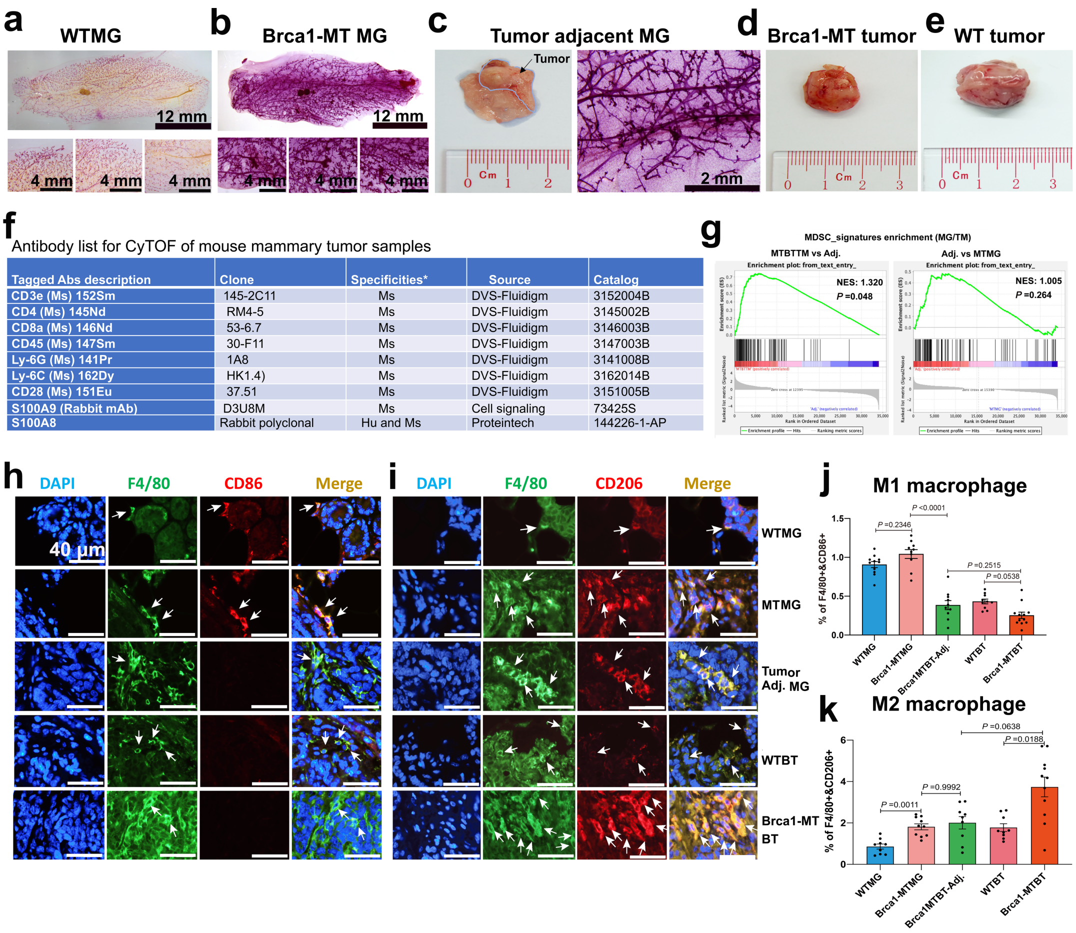


Antibody list for CyTOF of mouse mammary tumor samples

| Tagged Abs description | Clone | Specificities* | Source | Catalog |
|---|---|---|---|---|
| CD3e (Ms) 152Sm | 145-2C11 | Ms | DVS-Fluidigm | 3152004B |
| CD4 (Ms) 145Nd | RM4-5 | Ms | DVS-Fluidigm | 3145002B |
| CD8a (Ms) 146Nd | 53-6.7 | Ms | DVS-Fluidigm | 3146003B |
| CD45 (Ms) 147Sm | 30-F11 | Ms | DVS-Fluidigm | 3147003B |
| Ly-6G (Ms) 141Pr | 1A8 | Ms | DVS-Fluidigm | 3141008B |
| Ly-6C (Ms) 162Dy | HK1.4) | Ms | DVS-Fluidigm | 3162014B |
| CD28 (Ms) 151Eu | 37.51 | Ms | DVS-Fluidigm | 3151005B |
| S100A9 (Rabbit mAb) | D3U8M | Ms | Cell signaling | 73425S |
| S100A8 | Rabbit polyclonal | Hu and Ms | Proteintech | 144226-1-AP |

**Supplementary Figure 1. The tumor development processes in *Brca1$^{Co/Co}$;MMTV-Cre* mice, related to Fig. 1.**

**a-b** The wholemount image of the fourth mammary gland of 10-month-old Wild type (WT) mice (**a**) (n=6 mice), and Brca1 mutant (Brca1 MT) mice (**b**) (n=6 mice) stained with carmine alum. **c** The third mammary gland of 12-month-old *Brca1 co/co/MMTV* mice with a small tumor and a part of the tumor adjacent mammary gland stained with carmine alum (n=3 mice). **d-e** The wholemount image of mammary tumors from 12 months old *Brca1*-MT and WT mice (n=3 mice/group). **f** The list of the antibodies used in CyTOF analysis. **g** GSEA analysis of signature genes in tumor (TM) tissues of Brca1-MT breast tumor (BT) bearing mice (MTBTTM) vs tumor adjacent mammary tissues (Adj.) and Adj. vs Brca1-MT mammary tissues (MTMG). **h-i** M1 macrophages with co-staining of both F4/80 (green) and CD86 (red) antibodies and M2 macrophages with co-staining of both F4/80 and CD206 in the same cohort of mice in Fig. 1**a**. **j-k** Quantifications (**j**, 11 pictures from WTMG, 10 from MTMG, 10 from adj.MG, 10 from WTBT, 12 from MTBT) in (**h**) and (**k**, 9 pictures from WTMG, 10 from MTMG, 9 from adj.MG, 9 from WTBT, 11 from MTBT) in (**i**). The data are expressed as means ± SD (**j-k**) from 3-4 pictures per mice of 3 mice and P values determined by one-way ANOVA followed by Tukey's multiple comparisons (**j-k**), and permutation test (**g**). Scale bar (**h-i**): white color, 40 μM. Source data are provided as a Source data file.

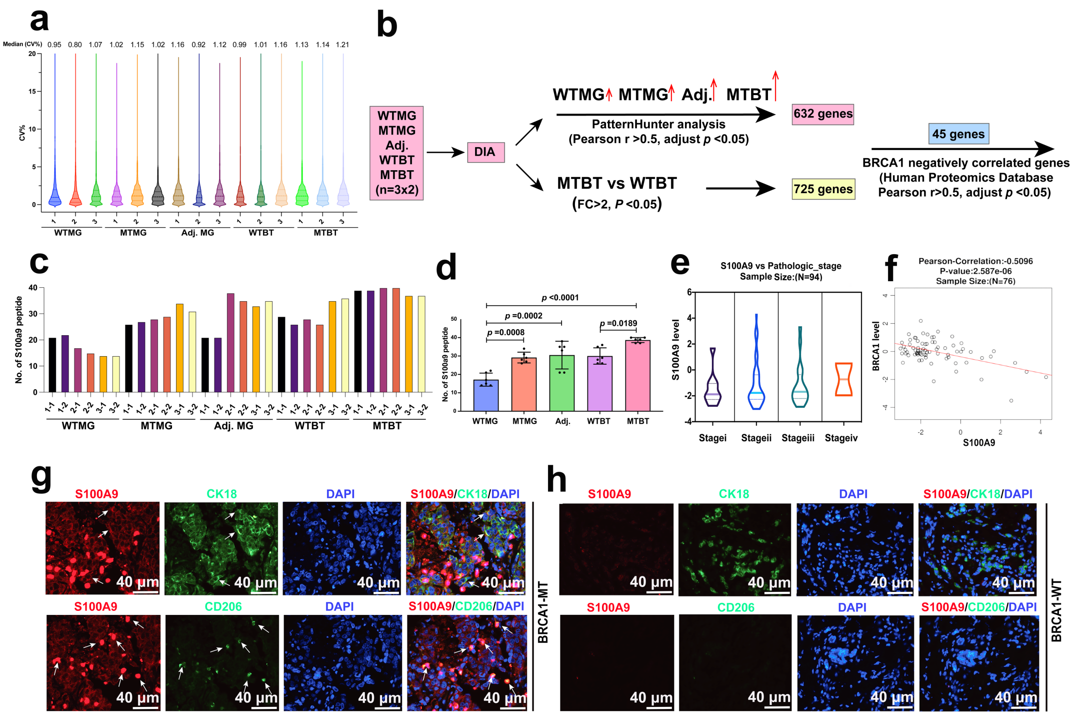


**Supplementary Figure 2. Up regulated S100a9 induced oncogenic actions and TME in Brca1-MT mammary tissues, related to Fig. 2.**

**a** Coefficient of variations (CV) % of duplicate of each sample from the same cohort of 15 samples in Fig. 2c. **b** Identification of *S100a8, S100a9, Pglyrp1 and Colgalt1* as the top candidate genes from DIA data analysis by overlapping with 632 genes ($r > 0.5$, adjust $p < 0.05$) which kept increase compared to the WTMG during tumorigenesis processes, 725 genes (FC >2, $P < 0.05$) which are upregulated in MTBT compared with WTBT, and 45 top upregulated genes which are negatively regulated by BRCA1. All candidates had good scores by the calculation with Spectronaut (FDR<1%). **c-d** Identified numbers of S100A9 peptides in each group (**c**), and statistics analysis (**d**, n=3 mice, duplicate of each mouse) from the same cohort of samples with duplicate injection for each sample in (**a**). **e-f** Increased S100A9 gene expression was positively correlated with increasing cancer stage by violin plot (**e**) and Pearson correlation of BRCA1 and S100A9 at protein level (-0.5096, p=2.587-e06) from Clinical Proteomics Tumor Analysis Consortium (CPTAC) for The Cancer Genomic Atlas (TCGA) database (**f**). **g-h** Co-staining of S100A9 with either CK18 or CD206 in either BRCA1-MT (**g**) or BRCA1-WT human tumor tissues of PDX model (**h**) (n=3/group). The data are expressed as means ± SD (**d**) and P values determined by one-way ANOVA followed by Tukey's multiple comparisons (**d**), and permutation test (**f**). Scale bar (**g-h**): white color, 40 µM. Source data are provided as a Source data file.

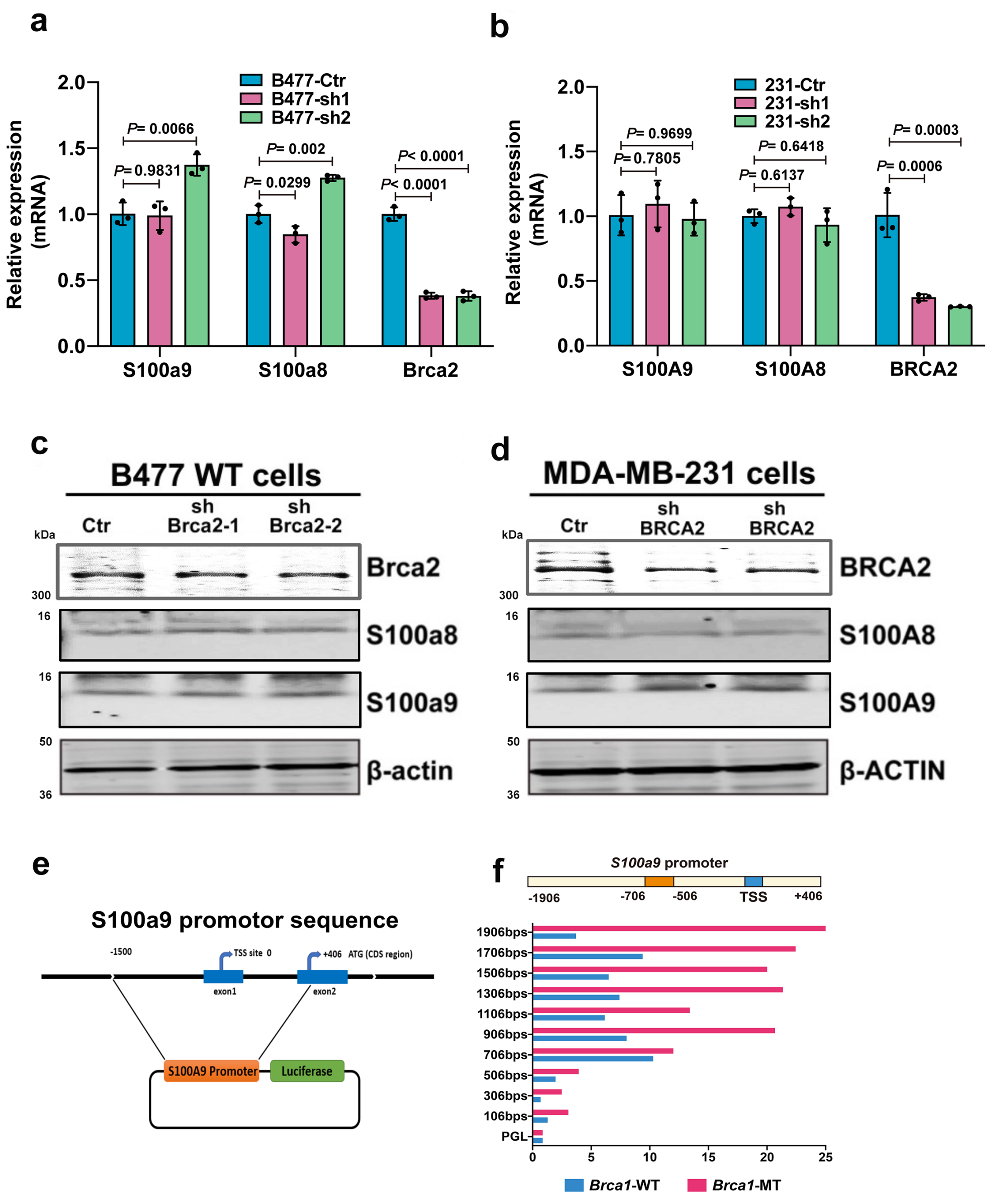


**Supplementary Figure 3. Regulation of S100a9 promoter.**

**a** Relative expression of *S100a8* and *S100a9* in B477 cells with expression of shBrca2-1 and shBrca2-2. **b** Relative expression of *S100a8* and *S100a9* in 231 cells expressing shBRCA2-1 and shBRCA2-2. **c** Protein level of S100a8 and S100a9 in B477 cells expressing shBrca2-1 and shBrca2-2. **d** Protein level of S100a8 and S100a9 in 231 cells expressing shBRCA2-1 and shBRCA2-2. **e** S100a9 promotor in LPG-luciferase construct. **f** Functional domains of DNA fragments (from -1906 bps to + 406 bps) of S100a9 promotor in mouse. The data are expressed as means ± SD (**a**, **b**) and P values determined by one-way ANOVA followed by Tukey's multiple comparisons. The experiments were independently repeated three times with similar results (**a-f**). Source data are provided as a Source data file.

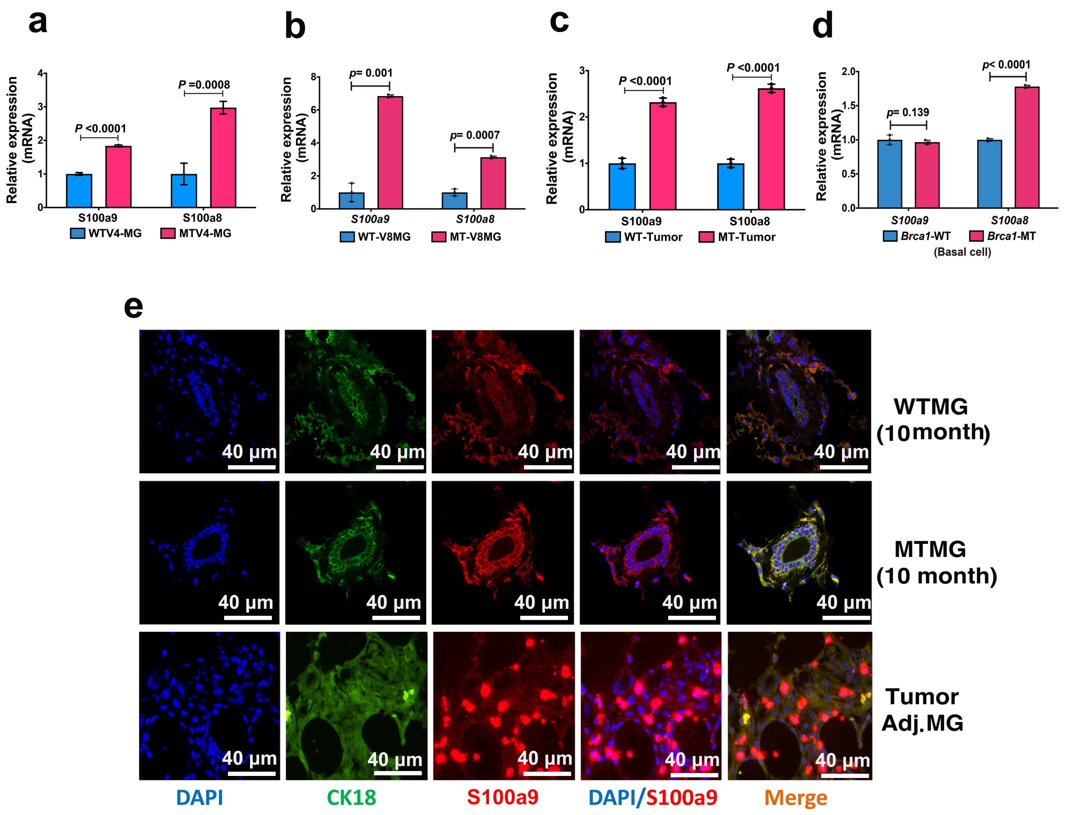


**Supplementary Figure 4. The expression and localization of S100a9/S100a8 in mammary tissues, related to Fig. 4.**

**a** S100a9/S100a8 mRNA expression in 4-month WT virgin mammary gland (WTV4MG), 4-month MT virgin mammary gland (MTV4MG) (n=3 mice). **b** S100a9/S100a8 mRNA expression by qPCR in WT 8-month virgin mammary gland (WTV8MG), MT 8-month virgin mammary gland (MTV8MG) (n=3 mice). **c** S100a9/S100a8 mRNA expression by qPCR in WT tumor (WT Tu), and MT tumor (MT Tu) tissues (n=3 mice). **d** S100a9/S100a8 mRNA expression in basal cell population. **e** Co-staining with S100a9 and CK18 antibodies by IF on 10-month WT mammary gland (WTMG), MT mammary gland (MTMG), and tumor adjacent mammary gland (Tumor Adj. MG) of Brca1 MT mice (n=3 pairs/group).The data are expressed as means ± SD (**a-d**) and P values determined by unpaired two-tailed Student's t test. The experiments were independently repeated three times with similar results (**d-e**). Scale bar: white color, 40 μM. Source data are provided as a Source data file.

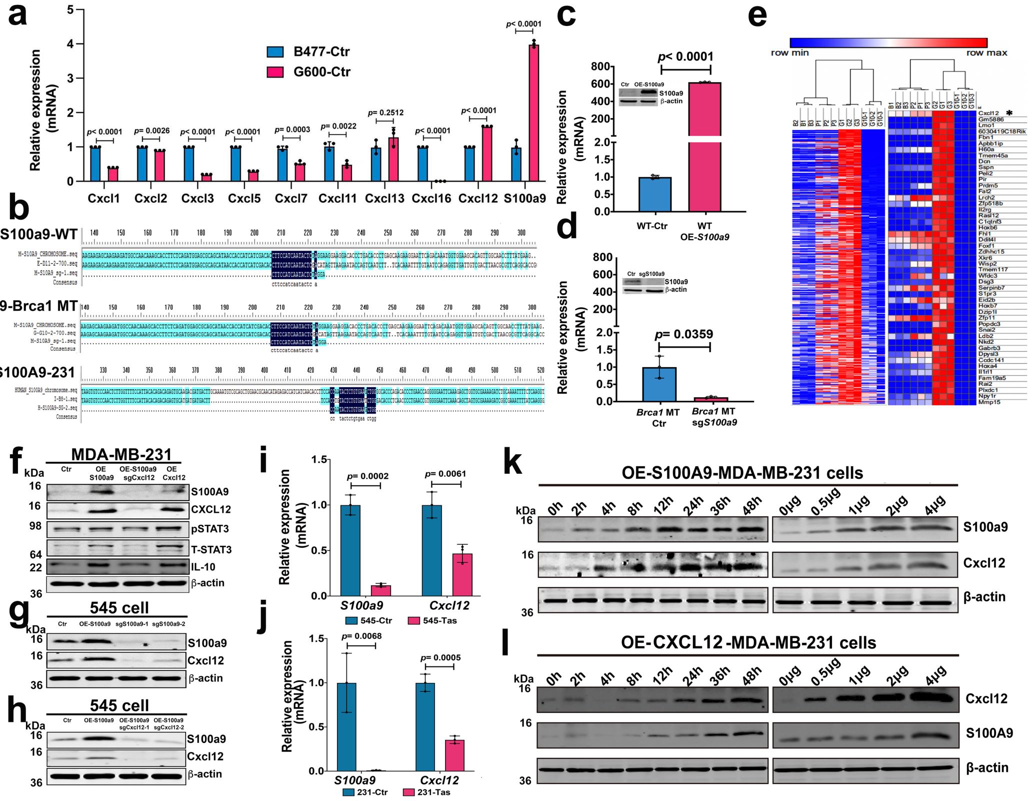


**Supplementary Figure 5. Positive regulation loop between S100a9 and Cxcl12 amplifies oncogenic signals in Brca1-MT epithelial cells Fig. 5.**

**a** mRNA expression of ten cytokines in B477 and G600 cells. **b** Sanger sequences from Brca1-WT, Brca1-MT, and MDA-MB-231 (231) cells with sg*S100A9*. **c-d** Expression of m*S100a9* at mRNA and protein levels in both B477and G600 cells expressing either OE-*S100a9* (**c**) or sg*S100a9* (**d**). **e** Gene expression files of RNA-sequence results from triplicated samples of B477 (B1-3), B477-OE-S100a9 (P1-3), G600 (G1-3), and G600-sgS100a9 (G-10:1-3) cells presented by heatmap. **f** Protein levels of S100a9, Cxcl12, pStat3 and Il10 in either OE-*S100A9*-231 cells, or sg*CXCL12*/OE-*S100A9*-231 cells, or OE-*CXCL12*-231 cells. **g** Protein levels of S100A9 and CXCL12 in 545 cells with over expression of *S100a9* (OE-S*100a9*) or expression of two different *S100a9* sgRNAs. **h** Protein levels of S100a9 and Cxcl12 in OE-*S100a9*-545 cells with the expression of two different *Cxcl12* sgRNAs. **i-j** Expressions of *S100a9* and *Cxcl12* in 545 (**i**) and 231(**j**) cells were treated, or sham treated with Tasquinimod. **k-l** Protein levels of S100a9 and Cxcl12 in 231 cells at different time course (0-48 hours) and different amount (0ug-4ug) after OE-S100a9 (**k**) or OE-Cxcl12 (**l**). The data are expressed as means ± SD (**a**, **c**, **d**, **i**, **j**) and P values determined by unpaired two-tailed Student's t test. The experiments were independently repeated three times with similar results (**a-l**). Source data are provided as a Source data file.

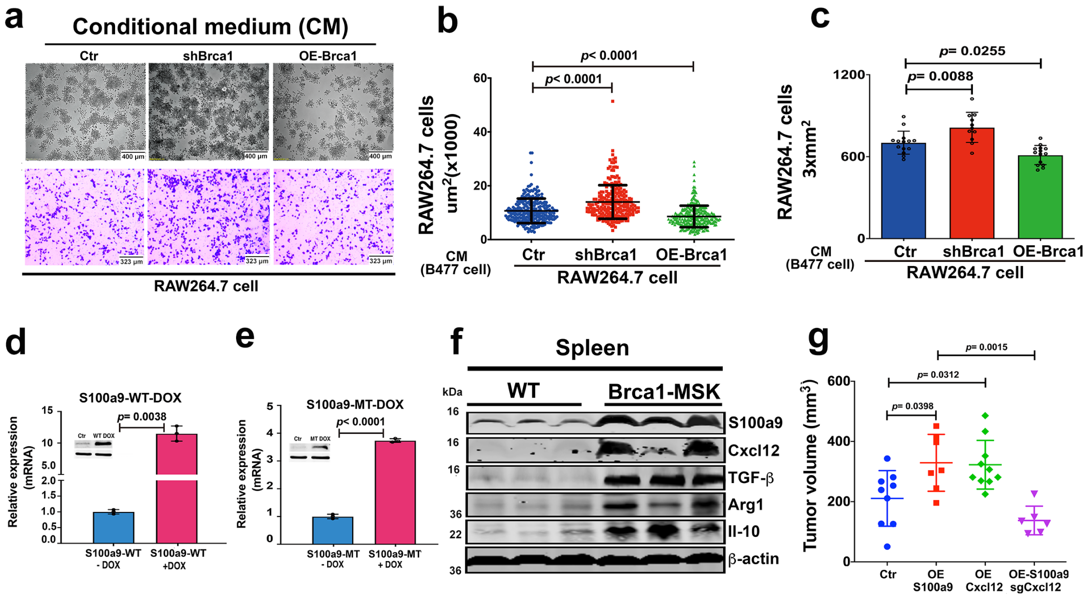


**Supplementary Figure 6. Expansion and activation of MDSCs induced by S100a9 in epithelial cells, related to Fig. 6.**

**a** The colony size of RAW264.7 cells (upper panels) and migrated RAW264.7 cells (lower panels) in different conditional medium from B477 cells (Ctr), with expression shBrca1 vector in B477 cells (shBrca1), OE-Brca1 vector in B477 cells (OE-Brca1). **b-c** Quantification (**b,** n=234-277 cell colony area from independently repeated three times, 250 from Ctr, 234 from shBrca1, 277 from OE-Brca1) for upper panels in (**a**) and (**c,** n=11-14 pictures from independently repeated three times, 14 from Ctr, 11 from shBrca1, 14 from OE-Brca1) for lower panels in (**a**). **d-e** mRNA and Protein levels of S100a9 in B477 (**d**) and G600 (**e**) cells after 48 hours induction with doxycycline (DOX) as determined by qPCR and Western blotting. **f** Protein levels of S100a9, Cxcl12, TGF-β, Arg1, and Il-10 in spleen tissues from 6-month WT and Brca1-MT mice (n=3 mice). **g** Tumor volume in Balb/c mice after implantation of EMT6 cells without (Ctr, n=9 mice), or with the expression of S100a9 (OE-S100a9, n=7 mice), Cxcl12 (OE-Cxcl12, n=10 mice), or sgCxcl12 in OE-S100a9 cells (n=6 mice). The data are expressed as means ± SD (**b-e, g**) and P values determined by unpaired two-tailed Student’s t test (**d-e**) and by one-way ANOVA followed by Tukey’s multiple comparisons (**b**, **c**, **g**). The experiments were independently repeated three times with similar results (**a-f**). The data in **b**, **c** are from 3-100 pictures per time of three independent replicates. Source data are provided as a Source data file.

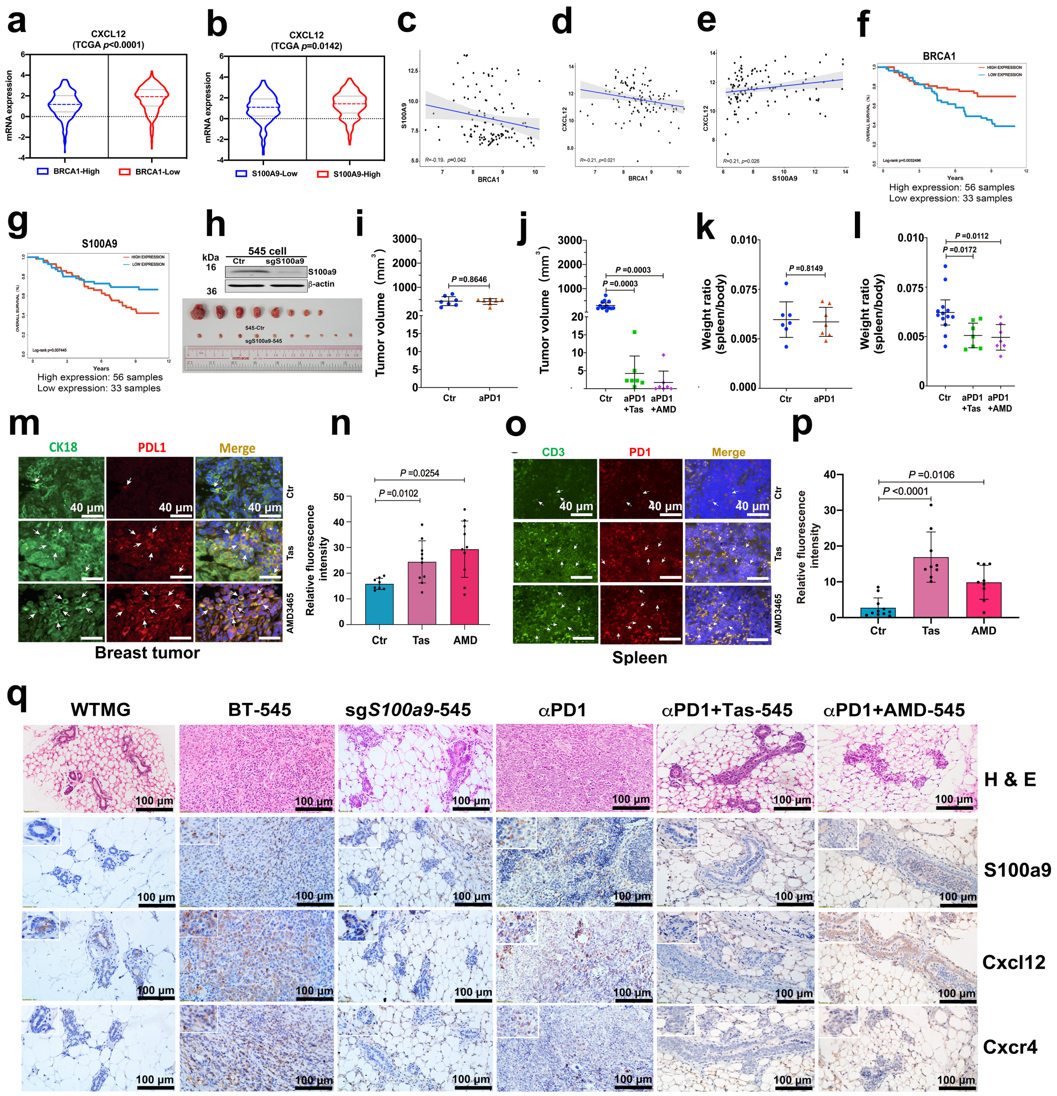


**Supplementary Figure 7. Combinatory treatments of drugs with aPD1 antibody inhibited tumor growth, related to Fig. 7.**

**a** CXCL12 expression in both BRCA1-low (n=366 samples) and BRCA1-high (n=467 samples) breast cancer patients from the TCGA datasets. **b** CXCL12 expression in both S100A9-low (n=201 samples) and S100A9-high (n=201 samples) breast cancer patients from the TCGA datasets. **c-e** Pearson correlation of S100A9 and BRCA1 (**c**), CXCL12 and BRCA1 (**d**), and S100A9 and CXCL12 (**e**) in breast cancer patients from the GSE19783-GPL6480 database (the gray areas show the 95% confidence interval, all n=216 in **c-e** ). **f-g** Overall survival curves of breast cancer patients with either low or high expression of BRCA1 (p=0.0032496) and the patients with low or high S100A9 expression from the GSE19783-GPL6480 database (p=0.007445). **h** Representative tumor images initiated with 545 cells in FVB mice ($4x10^6$ cells per fat pad, ctr: n=8 mice, sgS100a9: n= 10 mice). **i-l** Plots of tumor volume **(i-j)** and spleen weight ratio **(k-l)** in 545-FVB mouse model after treatment with αPD antibody only (n=7 mice), αPD +Tas (n=7 mice), and αPD +AMD (n=7 mice). **m-n** Representative images of mammary

tumor tissues treated with either Tas or AMD3465 only with antibodies against both CK18 (green) and PD-L1(red) (**m**) and quantification (**n**, 8 pictures from Ctr, 10 from Tas, 10 from AMD) in (**m**) (n=3 mice). **o-p** Representative images of spleen from same cohort of mice in (**m**) with antibodies against both CD3 (green) and PD1(red) (**o**) and quantification (**p**, 10 pictures from Ctr, 9 from Tas, 9 from AMD) in (**o**) (n=3 mice). **q** The histologic section analysis IHC staining with antibodies against to S100a9, Cxcl12, and Cxcr4 on primary tumor tissues (before treatment) and mammary tissues (after the treatment) with αPD1, αPD1+Tas, and αPD1+AMD from the same cohorts of mice in **(i-l)** (n=6 mice/group). The data are expressed as means ± SD (**i-p**) and P values determined by unpaired two-tailed Student's t-test (**i**, **k**) and by one-way ANOVA followed by Tukey's multiple comparisons (**j**, **l**, **n**, **p**). Scale bar: white color, 40 μM, black color, 100 μM. Source data are provided as a Source data file.

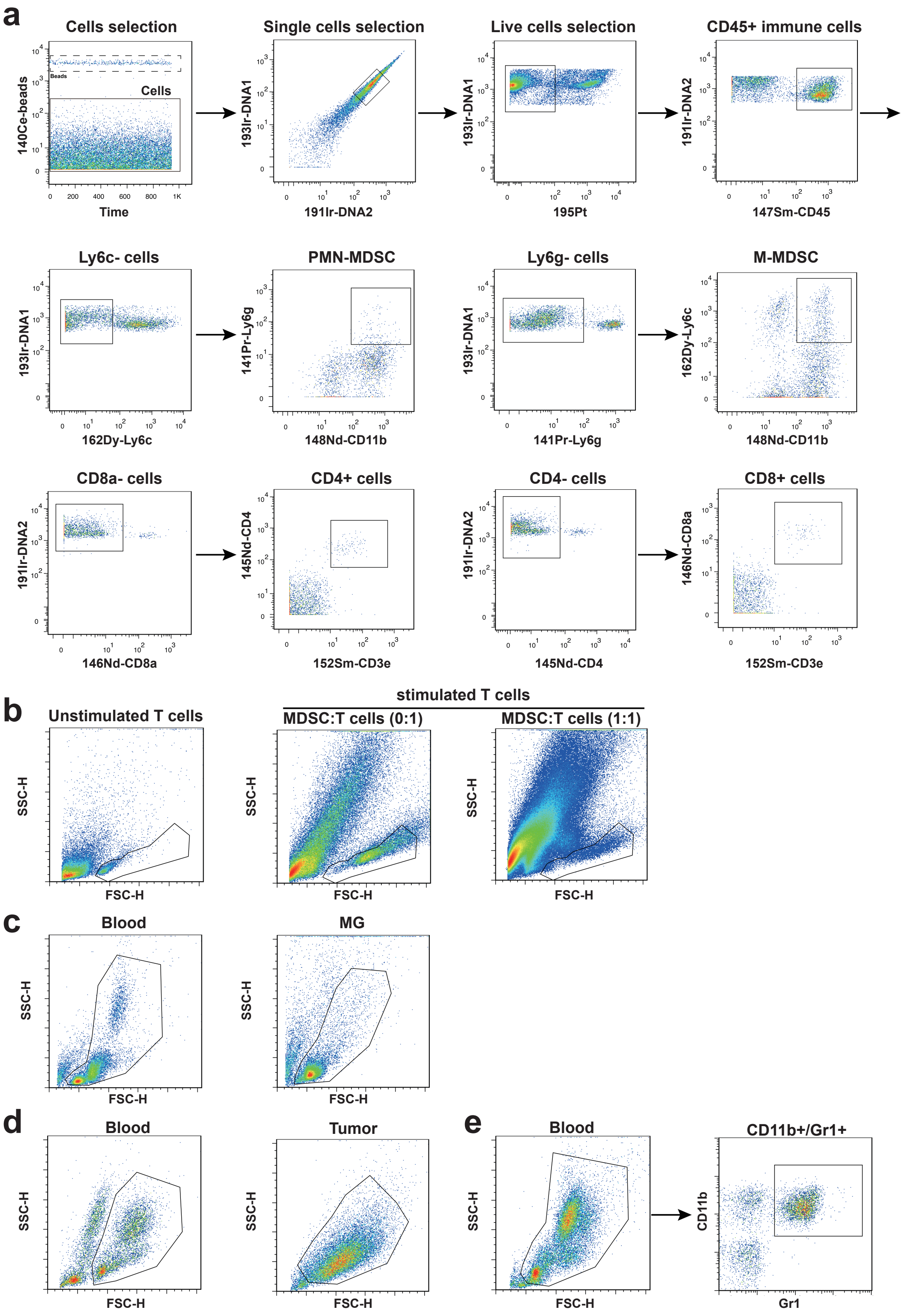


**Supplementary Figure 8. CyTOF and FACS gating strategies for cell analysis.**

**a** Cytof gating strategies for analyzing immune cell landscape in Figure 1b, 1c, 6q, 6r, 7d, 7q, 7r. **b** FACS gating strategies for analyzing T cell proliferation in Figure 1l, 1m, 1n, 1o. **c** FACS gating strategies for analyzing S100a9 and Arg1 positive cells in Figure 4d. **d** FACS gating strategies for analyzing S100a9 and CD11b/Gr1 positive cells in Figure 6m, 6n. **e** FACS gating strategies for analyzing CD11b/Gr1 positive cells in Figure 6o, 6p.

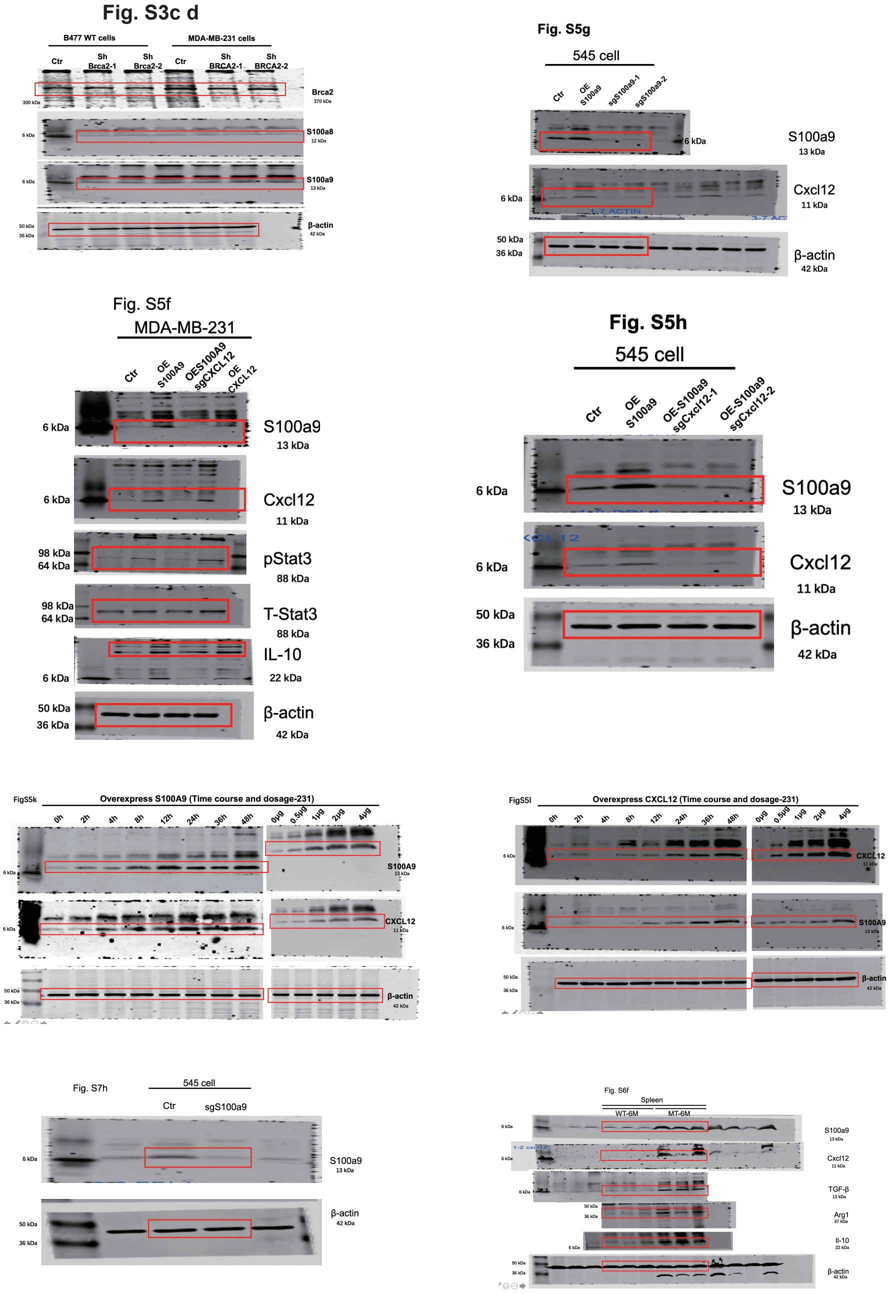


**Supplementary Figure 9. The uncropped and unprocessed gels for Western-Blot (WB) in Supplementary figures**

# Supplementary Table 1. DNA oligo sequences used in this study.

| Primers for qPCR | | |
|---|---|---|
| Gene | Forward Primer | Reverse primer |
| Human-*BRCA1* | GTCCCATCTGTCTGGAGTTGA | AAAGGACACTGTGAAGGCCC |
| Human-*S100A9* | CGGCTTTGACAGAGTGCAAG | GCCCCAGCTTCACAGAGTAT |
| Human-*S100A8* | TATCAGGAAAAAGGGTGCAGACG | TGCCACGCCCATCTTTATCA |
| Mouse-*Brca1* | GGAAATGGCAACTTGCCTAG | CTGCGAGCAGTCTTCAGAAAG |
| Mouse-*S100a9* | ATGGAGCGCAGCATAACCA | AAAGGTTGCCAACTGTGCTTC |
| Mouse-*S100a8* | TCAAGACATCGTTTGAAAGGAAA | TCTGCACAAACTGAGGACACT |
| Mouse-*Cxcl12* | AGAAAGCTTTAAACAAGGGGCG | AGAGGGAGGAGCGAGTTACA |
| Oligonucleotides | | |
| Human-sgS100A9 -1: | CACCGACTTGCAAAATGTCGCAGC | AAACGCTGCGACATTTTGCAAGTC |
| Human-sgS100A9 -2: | CACCGCCAATACTCTGTGAAGCTGG | AAACCCAGCTTCACAGAGTATTGGC |
| Mouse-sgS100a9 -1: | CACCGCTTCCATCAATACTCTAGGA | AAACTCCTAGAGTATTGATGGAAGC |
| Mouse sgS100a9 -2: | CACCGTCATCGACACCTTCCATCAA | AAACTTGATGGAAGGTGTCGATGAC |
| Mouse sgS100a9 -3: | CACCGACAAAGCACCTTCTCAGATG | AAACCATCTGAGAAGGTGCTTTGTC |
| Human sgCXCL12 -1: | CACCGcgccaaggtcgtggtcgtgc | AAACgcacgaccacgaccttggcgC |
| Human sgCXCL12 -2: | CACCggcagagcgcggtcagcacg | AAACcgtgctgaccgcgctctgcc |
| Human sgCXCL12 -3: | CACCgaccagcacgaccacgacct | AAACaggtcgtggtcgtgctggtc |
| Mouse sgCxcl12 -1: | CACCGcgccaaggtcgtcgccgtgc | AAACgcacggcgacgaccttggcgC |
| Mouse sgCxcl12 -2: | CACCggccagcacggcgacgacct | AAACaggtcgtcgccgtgctggcc |
| Mouse sgCxcl12 -3: | CACCggtcgtcgccgtgctggccc | AAACgggccagcacggcgacgacc |

# Supplementary Table 2. Software and website used in this study.

| | |
|---|---|
| GraphPad Prism software 8.0 | https://www.graphpad.com |
| Flow Jo_v10 | http://www.flowjo.com/ |
| Cytobank based viSNE | https://www.cytobank.org/ |
| ImageJ | https://imagej.nih.gov/ij/ |
| Morpheus-BroadInstitute | https://software.broadinstitute.org/morpheus/ |
| KEGG (Kyoto Encyclopedia of Genes and Genomes) | https://www.genome.jp/kegg/pathway |
| Principal component analysis (PCA) | https://www.metaboanalyst.ca/faces/home.xhtml |
