# Supplementary material for: S100A9-CXCL12 activation in BRCA1-mutant breast cancer promotes an immunosuppressive microenvironment associated with resistance to immunotherapy
Source: Nat Commun. 2022 Mar 18;13:1481. doi: 10.1038/s41467-022-29151-5 (PMC8933470; doi:10.1038/s41467-022-29151-5)
Supplement: Supplementary file 2 — Reporting Summary [file 41467_2022_29151_MOESM2_ESM.pdf]

nature research

Corresponding author(s): Xiaoling Xu
Last updated by author(s): Feb 21, 2022

Reporting Summary

Nature Research wishes to improve the reproducibility of the work that we publish. This form provides structure for consistency and transparency in reporting. For further information on Nature Research policies, see our Editorial Policies and the Editorial Policy Checklist.

Statistics

For all statistical analyses, confirm that the following items are present in the figure legend, table legend, main text, or Methods section.

n/a	Confirmed	
☐	☒	The exact sample size (*n*) for each experimental group/condition, given as a discrete number and unit of measurement
☐	☒	A statement on whether measurements were taken from distinct samples or whether the same sample was measured repeatedly
☐	☒	The statistical test(s) used AND whether they are one- or two-sided<br>*Only common tests should be described solely by name; describe more complex techniques in the Methods section.*
☒	☐	A description of all covariates tested
☒	☐	A description of any assumptions or corrections, such as tests of normality and adjustment for multiple comparisons
☐	☒	A full description of the statistical parameters including central tendency (e.g. means) or other basic estimates (e.g. regression coefficient) AND variation (e.g. standard deviation) or associated estimates of uncertainty (e.g. confidence intervals)
☐	☒	For null hypothesis testing, the test statistic (e.g. *F*, *t*, *r*) with confidence intervals, effect sizes, degrees of freedom and *P* value noted<br>*Give P values as exact values whenever suitable.*
☒	☐	For Bayesian analysis, information on the choice of priors and Markov chain Monte Carlo settings
☐	☒	For hierarchical and complex designs, identification of the appropriate level for tests and full reporting of outcomes
☐	☒	Estimates of effect sizes (e.g. Cohen's *d*, Pearson's *r*), indicating how they were calculated

Our web collection on statistics for biologists contains articles on many of the points above.

Software and code

Policy information about availability of computer code

Data collection	Image lab version 6.0.1(Bio-Rad),Image Studio Ver 5.2(LI-COR),LAS-V4.9(Leica),cellSens Dimension 1.18-Build 16686(Olympus)
Data analysis	GraphPad Prism 8.0 was used to generate bar plots, dot plots,curve plots,violin plots;Cytobank (7.3.0) and Flow Jo_v10 was used to analyze CyTOF and flow cytometry data;GSEA_4.1.0 was used to draw GSEA MDSC_signatures enrichment;MORPHEUS (https://software.broadinstitute.org/morpheus/)was used to draw heatmap;Venny2.0 was used to draw venny diagram;Kaplan-meier plotter(https://kmplot.com/analysis/index.php?p=service) was used to draw survival curve; ImageJ 1.46r was used to analyze IHC,IF and cell staining figures;Metaboanalyst online tool-MetaboAnalyst 5.0 (https://www.metaboanalyst.ca/) was used for PCA analysis and PatternHunter analysis;KEGG (Kyoto Encyclopedia of Genes and Genomes https://www.genome.jp/kegg/) and KOBAS 3.0(http://kobas.cbi.pku.edu.cn/kobas3/genelist/) was used to do pathway enrichment; the statistical significance was performed by GraphPad Prism 8.0. The raw RNA sequencing data were aligned to mouse genome reference mm10 by using hisat2 (v2.1.0) with default parameters. FeatureCounts (v1.6.3) was applied to count the number of reads that mapping to each genes.

For manuscripts utilizing custom algorithms or software that are central to the research but not yet described in published literature, software must be made available to editors and reviewers. We strongly encourage code deposition in a community repository (e.g. GitHub). See the Nature Research guidelines for submitting code & software for further information.

Data

Policy information about availability of data

All manuscripts must include a data availability statement. This statement should provide the following information, where applicable:

- Accession codes, unique identifiers, or web links for publicly available datasets
- A list of figures that have associated raw data
- A description of any restrictions on data availability

All data supported the paper are present in the paper and/or the Supplementary Materials. The original datasets are also available from the corresponding author upon request. The RNA-seq data have been deposited at Sequence Read Archive (SRA) database with following access numbers: PRJNA766531 [https://www.ncbi.nlm.nih.gov/bioproject/PRJNA766531/], PRJNA719246 [https://www.ncbi.nlm.nih.gov/bioproject/PRJNA719246/] and PRJNA719077 [https://www.ncbi.nlm.nih.gov/bioproject/PRJNA719077/]. The DIA-MS proteomics data have been deposited at PRoteomics IDEntifications Database (PRIDE) with following access numbers: PXD030328 (DIA raw data) [http://www.ebi.ac.uk/pride/archive/projects/PXD030328] and PXD030355 (DDA raw data) [http://www.ebi.ac.uk/pride/archive/projects/PXD030355]. Breast cancer data from The Cancer Genomic Atlas (TCGA-BRCA.sampleMap/HiSeqV2) database and GSE19783-GPL6480 dataset was used. Source data are provided with this paper. The remaining data are available within the Article, Supplementary Information or Source Data file.

Field-specific reporting

Please select the one below that is the best fit for your research. If you are not sure, read the appropriate sections before making your selection.

[x] Life sciences [ ] Behavioural & social sciences [ ] Ecological, evolutionary & environmental sciences

For a reference copy of the document with all sections, see nature.com/documents/nr-reporting-summary-flat.pdf

Life sciences study design

All studies must disclose on these points even when the disclosure is negative.

Sample size	We used sample sizes containing 3 or more biological replicates which can provide adequate statistical power in biochemical analysis. In addition, for both in vitro and in vivo experiments, the group sizes were also selected empirically based upon our prior knowledge of responses of these cells and animals. We have described the exact sample sizes and their statistic significance for each experiment in our manuscript.
Data exclusions	No data were exclude from the analyses.
Replication	All experiments were independently repeated as indicated and were reliably reproduced(described in figure legends for further details).
Randomization	All mice were randomly allocated into different group according experiment design.
Blinding	Blinding experiments were not performed as we need to know genotypes of cell lines or mouse models, i.e. wild type or mutant. Among each genotype, we randomly divided the cells or animal models into several groups and treat them using different conditions in blinding way. We used enough number of sample sizes or animals to avoid unbiased result.

Reporting for specific materials, systems and methods

We require information from authors about some types of materials, experimental systems and methods used in many studies. Here, indicate whether each material, system or method listed is relevant to your study. If you are not sure if a list item applies to your research, read the appropriate section before selecting a response.

Materials & experimental systems

n/a	Involved in the study	
[ ]	[x]	Antibodies
[ ]	[x]	Eukaryotic cell lines
[x]	[ ]	Palaeontology and archaeology
[ ]	[x]	Animals and other organisms
[x]	[ ]	Human research participants
[x]	[ ]	Clinical data
[x]	[ ]	Dual use research of concern

Methods

n/a	Involved in the study	
[x]	[ ]	ChIP-seq
[ ]	[x]	Flow cytometry
[x]	[ ]	MRI-based neuroimaging

Antibodies

Antibodies used	Anti-CD11B (M1/70, 101211), anti-CD3(17A2, 100203), anti-Gr1(RB6-8C5, 108419), anti-CD28(37.51, 102109), anti-CD4(GK1.5, 100405) antibodies were from Biolegend. Anti-CD8a (53-6.7, 42-0081-82) was from Invitrogen. Anti-Arg1(IC5868A) was from R&D.

Anti-S100a9(PE, D3U8M), anti-S100a9(#73425), anti- p-Stat3(4113), anti-Cyclin-D1(2978S), anti-PD1 (D7D5W), F4/80 (D2S9R) and anti-PDL1 (E1L3N)antibodies were from Cell Signaling. Anti-Arg1(ab60176), anti-CD11B(ab8878), anti- CXCR7(ab117836), anti-IL-10 (ab189392), anti- CXCR4(ab124824) antibodies were from Abcam. Anti-BRCA1(SC-642), anti-c-Myc(SC-764), anti-TGF-β1(SC-130348), Bcl-XL(SC-634), anti-CK18(sc-53256) were from Santa Cruz. Anti-S100A8(157921-1-AP) was from Proteintech. Anti-CXCL12 (MA5-23759) was from ThermoFisher. Anti-CD206 (AF2535) was from bio-techne. Anti-CD86 (LS-C392134) was from LSBio.

Validation

All antibodies are from commercial sources and the validation data are available on website of the manufactures. The applicaton of all antibodies were followed by the instructions of the website.
Anti-CD11B(1:100 for FC),clone M1/70, Biolegend (101211):https://www.biolegend.com/en-us/products/apc-anti-mouse-human-cd11b-antibody-345
anti-CD3 (1:100 for FC), clone 17A2, Biolegend (100203): https://www.biolegend.com/en-us/products/fitc-anti-mouse-cd3-antibody-45
anti-Gr1 (1:100 for FC), clone RB6-8C5, Biolegend (108419): https://www.biolegend.com/en-us/products/alexa-fluor-488-anti-mouse-ly-6g-ly-6c-gr-1-antibody-2711
anti-CD28 (1:100 for FC), clone 37.51, Biolegend (102109):https://www.biolegend.com/en-us/products/apc-anti-mouse-cd28-antibody-112
anti-CD4 (1:100 for FC), clone GK1.5, Biolegend (100405): https://www.biolegend.com/en-us/products/fitc-anti-mouse-cd4-antibody-248
Anti-CD8a (1:100 for FC), clone 53-6.7,Invitrogen (42-0081-82):https://www.thermofisher.com/antibody/product/CD8a-Antibody-clone-53-6-7-Monoclonal/42-0081-82
Anti-Arg1(1:100 for FC), R&D (IC5868A):https://www.rndsystems.com/products/human-mouse-arginase-1-arg1-apc-conjugated-antibody_ic5868a
Anti-S100a9-PE(1:100 for FC), clone D3U8M, Cell Signaling(93941):https://www.cellsignal.com/products/antibody-conjugates/s100a9-d3u8m-rabbit-mab-rodent-specific-pe-conjugate/93941
Anti-S100a9 (1:300 for WB), clone D3U8M, Cell Signaling (73425):https://www.cellsignal.com/products/primary-antibodies/s100a9-d3u8m-rabbit-mab-rodent-specific/73425
Anti-p-Stat3 (1:1000 for WB), clone M9C6, Cell Signaling (4113):https://www.cellsignal.com/products/primary-antibodies/phospho-stat3-tyr705-m9c6-mouse-mab/4113
Anti-Cyclin-D1 (1:1000 for WB), clone 92G2, Cell Signaling (2978S):https://www.cellsignal.com/products/primary-antibodies/cyclin-d1-92g2-rabbit-mab/2978
Anti-F4/80 (1:50 for IF), clone D2S9R, Cell Signaling (70076):https://www.cellsignal.com/products/primary-antibodies/f4-80-d2s9r-xp-rabbit-mab/70076
Anti-PD1(1:50 for IF), clone D7D5W, Cell Signaling (84651):https://www.cellsignal.com/products/primary-antibodies/pd-1-intracellular-domain-d7d5w-xp-rabbit-mab/84651
Anti-PDL1(1:50 for IF), clone E1L3N, Cell Signaling (13684):https://www.cellsignal.com/products/primary-antibodies/pd-l1-e1l3n-xp-rabbit-mab/13684
Anti-CD3 (1:100 for IF), Abcam(ab5690):https://www.abcam.com/cd3-antibody-ab5690.html
Anti-Arg1(1:1000 for WB), Abcam (ab60176):https://www.abcam.com/liver-arginase-antibody-ab60176.html
Anti-CXCR7(1:1000 for WB), Abcam (ab117836):https://www.abcam.com/gpcr-rdc1cxcr-7-antibody-ab117836.html
Anti-IL10(1:1000 for WB), clone JES5-2A5, Abcam (ab189392):https://www.abcam.com/il-10-antibody-jes5-2a5-ab189392.html
Anti-CXCR4(1:100 for IHC) , clone UMB2,Abcam (ab124824):https://www.abcam.com/products?keywords=124824
Anti-BRCA1(1:200 for WB), clone C-20,Santa Cruz (SC-642):https://www.scbt.com/p/brca1-antibody-c-20?requestFrom=search
Anti-c-Myc(1:200 for WB),clone N262, Santa Cruz (SC-764):https://www.scbt.com/p/c-myc-antibody-n-262?requestFrom=search
Anti-TGF-β1(1:300 for WB), clone 3C11, Santa Cruz (SC-130348):https://www.scbt.com/p/tgf-beta1-antibody-3c11
Anti-Bcl-XL(1:500 for WB), clone S-18 , Santa Cruz (SC-634):https://www.scbt.com/p/bcl-xs-l-antibody-s-18?requestFrom=search
Anti-CK18(1:50 for IF), clone LDK18, Santa Cruz (sc-53256):https://www.scbt.com/p/cytokeratin-18-antibody-ldk18?requestFrom=search
Anti-S100A8(1:500 for WB, 1:50 for IHC), Proteintech(157921-1-AP):https://www.ptglab.com/products/S100A8-Antibody-15792-1-AP.htm
Anti-CXCL12(1:300 for WB, 1:50 for IHC), clone 79018, ThermoFisher(MA5-23759):https://www.thermofisher.com/antibody/product/CXCL12-Antibody-clone-79018-Monoclonal/MA5-23759
Anti-CD206(1:100 for IF), bio-techne(AF2535):https://www.bio-techne.com/p/antibodies/mouse-mmr-cd206-antibody_af2535
Anti-CD86(1:50 for IF), LSBio (LS-C392134):https://www.lsbio.com/targets/cd86/g4600?adid=10122&prefix=a&gclid=EAIaIQobChMIz9Tkotb39QIVUaSWCh3WVAtnEAAYAiAAEgJAWPD_BwE#SearchTable_div

Eukaryotic cell lines

Policy information about cell lines

Cell line source(s)

The immortalized mouse Brca1-WT epithelial cell line B477 was derived from the mammary gland of Brca1-WT mice(Brca1+/+;p53+/-);The mouse Brca1-mutant epithelial cell line G600 was derived from the mammary gland of Brca1-MT mice (Brca1Δexon11/Δexon11;p53+/-). The breast tumor cell line 545 was derived from Brca1 co/co MMTV tumor mice.The EMT6 cell line, MDA-MB-231 cell line, and the 293T cell line were from American Tissue Culture Collection(ATCC).

Authentication

Morphology check by microscope was done for each cell line for authentication.

Mycoplasma contamination

All cell lines tested negative for Mycoplasma contamination.

Commonly misidentified lines (See ICLAC register) | None.

Animals and other organisms

Policy information about studies involving animals; ARRIVE guidelines recommended for reporting animal research

Laboratory animals: Mixed background of FVB/129SvEv/Black Swiss female mice of 2 months, 4 months, 6 months, 8 months, 10 months;BALB/C, FVB and nude female mice of 6-8 weeks. All animal work performed in this study was approved by the University of Macau Animal Ethics Committee. Animals were maintained under a 12-hour light/12-hour dark cycle in a specific pathogen-free (SPF) animal facility with free access to water and a standard mouse diet. Room temperatures are maintained within the range of 23 ± 3 ℃. The Humidity levels are controlled globally, and it is maintained between 40-70%. All animal studies were conducted on female mice, and the procedures were performed under pentobarbital sodium anesthesia.

Wild animals: No wild animals were involved in the study.

Field-collected samples: The study did not involve samples collected from the field.

Ethics oversight: All experiments were approved by the Animal Ethics Committees of the Faculty of Health Science, University of Macau (Protocol ID: UMARE-AMEND-100).

Note that full information on the approval of the study protocol must also be provided in the manuscript.

Flow Cytometry

Plots

Confirm that:

- [x] The axis labels state the marker and fluorochrome used (e.g. CD4-FITC).
- [x] The axis scales are clearly visible. Include numbers along axes only for bottom left plot of group (a 'group' is an analysis of identical markers).
- [x] All plots are contour plots with outliers or pseudocolor plots.
- [x] A numerical value for number of cells or percentage (with statistics) is provided.

Methodology

Sample preparation: Single cells were collected and washed once with PBS. Approximately 5% of the total cells were used as negative controls, and the remaining cells were used for the experiment. The remaining cells were resuspended in 100 µl of PBS, mixed gently, and incubated with 1 µl of antibody on ice for 30 min in darkness. The cells were washed once with 1 ml of PBS and centrifuged for 5 min at 300 x g at 4°C. The supernatant was discarded, and the cell pellet was resuspended in 100 µl of PBS.The resuspended cells were then incubated with a secondary antibody (1:100) on ice for 30 min, washed once, and brought to a final volume of 200 µl with PBS. The solution was passed through a 40 µm filter, and FACS was performed.

Instrument: BD FACSCalibur

Software: CellQUEST Pro

Cell population abundance: The abundance of cell population is determined by cell markers and the details are described in Supplementary Information.

Gating strategy: The gating strategy is described in Supplementary Information-supplementary figure 8.

- [x] Tick this box to confirm that a figure exemplifying the gating strategy is provided in the Supplementary Information.
