# Supplementary material for: S100A9-CXCL12 activation in BRCA1-mutant breast cancer promotes an immunosuppressive microenvironment associated with resistance to immunotherapy
Source: Nat Commun. 2022 Mar 18;13:1481. doi: 10.1038/s41467-022-29151-5 (PMC8933470; doi:10.1038/s41467-022-29151-5)
Supplement: Supplementary file 4 — Description of Additional Supplementary Files [file 41467_2022_29151_MOESM4_ESM.pdf]

Title: Supplementary Data 1.

Description: RNA-seq. of CD11B+/GR1+ cells from MG, SP and BT between WT and MT mice.

Title: Supplementary Data 2.

Description: DIA-MS profile at different development groups from both Brca1 MT and WT mice.

Title: Supplementary Data 3.

Description: The CV% of duplicate per sample in DIA-MS.

Title: Supplementary Data 4.

Description: 453 genes in terms of its relative fold change from RNA sequencing on four mammary epithelial cell lines with differential expression of S100a9, including the WT, over expression (OE)-S100a9-WT, Brca1-MT, and sgS100a9-Brca1-MT cell lines
